# HIV-1 Vpr Accelerates Viral Replication during Acute Infection by Exploitation of Proliferating CD4^+^ T Cells *In Vivo*

**DOI:** 10.1371/journal.ppat.1003812

**Published:** 2013-12-05

**Authors:** Kei Sato, Naoko Misawa, Shingo Iwami, Yorifumi Satou, Masao Matsuoka, Yukihito Ishizaka, Mamoru Ito, Kazuyuki Aihara, Dong Sung An, Yoshio Koyanagi

**Affiliations:** 1 Laboratory of Virus Pathogenesis, Institute for Virus Research, Kyoto University, Kyoto, Kyoto, Japan; 2 Center for Emerging Virus Research, Institute for Virus Research, Kyoto University, Kyoto, Kyoto, Japan; 3 Department of Biology, Faculty of Sciences, Kyushu University, Fukuoka, Fukuoka, Japan; 4 Precursory Research for Embryonic Science and Technology (PRESTO), Japan Science and Technology Agency (JST), Kawaguchi, Saitama, Japan; 5 Laboratory of Viral Control, Institute for Virus Research, Kyoto University, Kyoto, Kyoto, Japan; 6 Department of Intractable Diseases, National Center for Global Health and Medicine, Shinjuku-ku, Tokyo, Japan; 7 Central Institute for Experimental Animals, Kawasaki, Kanagawa, Japan; 8 Institute of Industrial Science, The University of Tokyo, Meguro-ku, Tokyo, Japan; 9 Graduate School of Information Science and Technology, The University of Tokyo, Meguro-ku, Tokyo, Japan; 10 Division of Hematology-Oncology, University of California, Los Angeles (UCLA), Los Angeles, California, United States of America; 11 School of Nursing, UCLA, Los Angeles, California, United States of America; 12 AIDS Institute, UCLA, Los Angeles, California, United States of America; University of Massachusetts Medical School, United States of America

## Abstract

The precise role of viral protein R (Vpr), an HIV-1-encoded protein, during HIV-1 infection and its contribution to the development of AIDS remain unclear. Previous reports have shown that Vpr has the ability to cause G_2_ cell cycle arrest and apoptosis in HIV-1-infected cells *in vitro*. In addition, *vpr* is highly conserved in transmitted/founder HIV-1s and in all primate lentiviruses, which are evolutionarily related to HIV-1. Although these findings suggest an important role of Vpr in HIV-1 pathogenesis, its direct evidence *in vivo* has not been shown. Here, by using a human hematopoietic stem cell-transplanted humanized mouse model, we demonstrated that Vpr causes G_2_ cell cycle arrest and apoptosis predominantly in proliferating CCR5^+^ CD4^+^ T cells, which mainly consist of regulatory CD4^+^ T cells (Tregs), resulting in Treg depletion and enhanced virus production during acute infection. The Vpr-dependent enhancement of virus replication and Treg depletion is observed in CCR5-tropic but not CXCR4-tropic HIV-1-infected mice, suggesting that these effects are dependent on the coreceptor usage by HIV-1. Immune activation was observed in CCR5-tropic wild-type but not in *vpr*-deficient HIV-1-infected humanized mice. When humanized mice were treated with denileukin diftitox (DD), to deplete Tregs, DD-treated humanized mice showed massive activation/proliferation of memory T cells compared to the untreated group. This activation/proliferation enhanced CCR5 expression in memory CD4^+^ T cells and rendered them more susceptible to CCR5-tropic wild-type HIV-1 infection than to *vpr*-deficient virus. Taken together, these results suggest that Vpr takes advantage of proliferating CCR5^+^ CD4^+^ T cells for enhancing viremia of CCR5-tropic HIV-1. Because Tregs exist in a higher cycling state than other T cell subsets, Tregs appear to be more vulnerable to exploitation by Vpr during acute HIV-1 infection.

## Author Summary

HIV-1 encodes nine genes, five of which (*gag*, *pol*, *env*, *tat*, and *rev*) are essential for viral replication, and four, termed accessory genes (*vif*, *vpu*, *nef*, and *vpr*), appear to aid virus infection. Of the four accessory proteins, Vpr is the most enigmatic. It is well known that Vpr has the potential to cause G_2_ cell cycle arrest and apoptosis *in vitro*. Moreover, it has been reported that Vpr-mediated G_2_ arrest increases HIV-1 production *in vitro*. However, the role of Vpr in HIV-1 propagation *in vivo* remains unclear. Here, by using a humanized mouse model, we demonstrate that Vpr enhances CCR5-tropic but not CXCR4-tropic HIV-1 replication *in vivo* by exploiting Tregs during acute infection. In CCR5-tropic HIV-1-infected humanized mice, Vpr-dependent G_2_ cell cycle arrest and apoptosis are predominantly observed in infected Tregs, and wild-type but not *vpr*-deficient HIV-1-infected mice displayed acute Treg depletion. This Vpr-dependent Treg depletion may lead to immune activation and provide a pool of activated/proliferating CD4^+^ T cells, which supports subsequent HIV-1 expansion *in vivo*. This is the first report demonstrating the role of Vpr in HIV-1 infection *in vivo*.

## Introduction

Human immunodeficiency virus type 1 (HIV-1), the causative agent of acquired immunodeficiency syndrome (AIDS), encodes four viral accessory proteins: Vif, Vpu, Nef, and Vpr. Vpr is a small (96 amino acids) but multipotent protein which is known to induce G_2_ cell cycle arrest, apoptosis, and the enhancement of HIV-1 long terminal repeat (LTR)-driven transcription in infected cells [1]. Previous *in vitro* studies have reported that *vpr*-deficient HIV-1 is less replicative in CD4^+^ T cell lines [2] and cycling primary CD4^+^ T cells [3]. On the other hand, *vpr* deficiency modestly affects viral replication kinetics in tonsil histocultures in which resting CD4^+^ T cells dominantly reside [4]. *In vivo*, *vpr*-deficient SIV is less replicative but induces AIDS in macaque monkeys [5]. However, although the underlying molecular mechanisms of Vpr function have been widely investigated, the significance and the precise role(s) of Vpr *in vivo* remain unclear.

The main target of HIV-1 *in vivo* is CD4^+^ T cells. Based on their function and phenotype, primary CD4^+^ T cells are classified into three subsets: naive CD4^+^ T cells (Tns), memory CD4^+^ T cells (Tms), and regulatory CD4^+^ T cells (Tregs). It is speculated that such phenotypic and functional differences among these subsets closely associates with the infectivitiy, productivity, and replicativity of HIV-1 [6]. However, since cultured primary CD4^+^ T cell subsets do not retain all of their *in vivo* attributes, the dynamics of each subset on HIV-1 infection are poorly understood.

Among the CD4^+^ T cell subsets, Tregs constitute 5–10% of all CD4^+^ T cells in human, monkey, and mouse species [7]. The potential and phenotype of Tregs are under the control of a transcription factor called forkhead box P3 (FOXP3), which is exclusively expressed in Tregs [8]. Tregs are more actively proliferating *in vivo* than the other CD4^+^ T cell subsets [9]–[11]. It is well known that Tregs play a central role in the maintenance of self-tolerance and immune homeostasis [7]. In addition, it is implicated that Tregs are closely associated with immunopathological events such as autoimmune diseases [7] and infectious diseases [12]–[14]. In particular, there are lines of reports showing that HIV-1/SIV infection decreases Tregs in HIV-1-infected patients [15]–[17] and simian immunodeficiency virus (SIV)-infected macaque monkeys [18]–[20].

In this study, we infect a human hematopoietic stem cell (HSC)-transplanted humanized mouse model [21]–[25] with wild-type (WT) and *vpr*-deficient HIV-1 and investigate the fundamental role of Vpr in HIV-1 infection *in vivo*. Our findings suggest that Vpr plays a crucial role in accelerating CCR5-tropic (R5) but not CXCR4-tropic (X4) HIV-1 propagation during acute infection by utilizing CCR5^+^ proliferating CD4^+^ T cells including Tregs.

## Results

### Tregs are depleted during the acute phase of R5 HIV-1 infection

**Figure 1:**
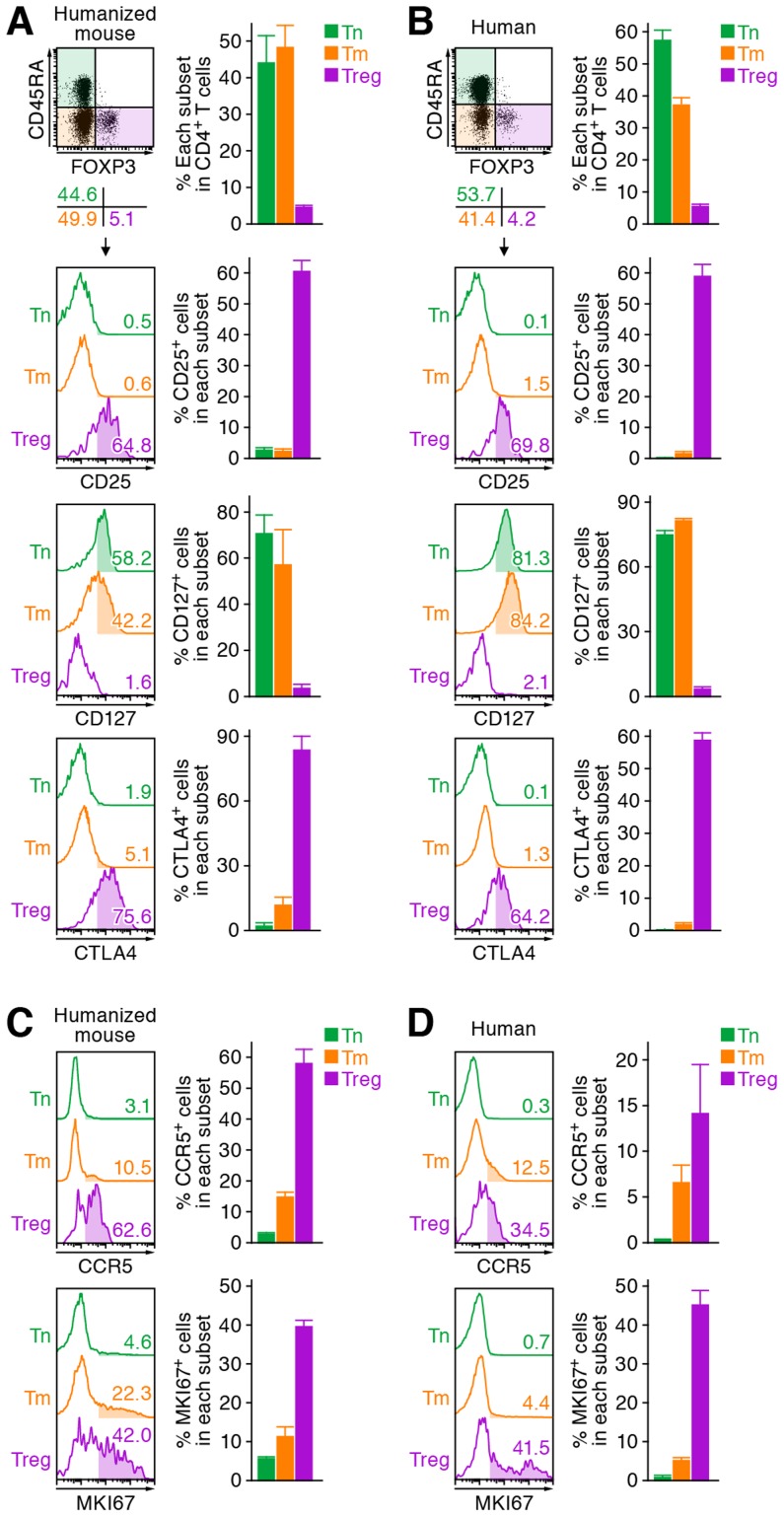
Comparison of the profile of CD4^+^ T cell subsets between human and humanized mouse. Human CD4^+^ T cells isolated from the spleen of humanized mice (A and C, n = 8) and the PB of HIV seronegative humans (B and D, n = 6) and were classified into Tn (CD45^+^ CD3^+^ CD4^+^ CD45RA^+^ FOXP3^−^ cells), Tm (CD45^+^ CD3^+^ CD4^+^ CD45RA^−^ FOXP3^−^ cells), and Treg (CD45^+^ CD3^+^ CD4^+^ CD45RA^−^ FOXP3^+^ cells) by flow cytometry. Representative dot plots and histograms are shown on the left panels. The percentage of each subset in CD4^+^ T cells (A and B, top) and the percentages of the cells positive for CD25, CD127, CTLA4, CCR5, and MKI67 in each subset are respectively shown on the right panels. In the left panels, the numbers under the dot plots (A and B, top) indicate the percentage of the cells in each quadrant, and the numbers in each histogram indicate the positivity. Data represent mean ± SEM.

We first characterized the profile of human CD4^+^ T cell subsets, including Tns, Tms, and Tregs, in human peripheral blood mononuclear cells (PBMCs) isolated from HIV-1-negative healthy donors and in the spleen of humanized mice [21]–[23]. As shown in Figure 1A, we detected 6.3±0.2% FOXP3^+^ CD4^+^ T cells in splenic human CD4^+^ T cells of humanized mice, which was comparable to those in human peripheral CD4^+^ T cells (5.4±0.6%; Figure 1B). Consistent with previous reports [26]–[29], we also confirmed that the phenotypes of Tregs including the expression levels of CD25, CD127, and cyototoxic T-lymphocyte associated protein 4 (CTLA4; also known as CD152) in humanized mice (Figure 1A) were similar to those in humans (Figure 1B). Since the suppressive function of the Tregs differentiated in humanized mouse models has been demonstrated previously [26]–[29], our results strongly suggest that the majority of FOXP3^+^ CD4^+^ T cell population in our humanized mouse model is Tregs. Moreover, the expression level of CCR5, an HIV-1 coreceptor, was higher on Tregs than on Tms and Tns in both humans and humanized mice (Figure 1C and 1D). Furthermore, in line with previous studies reporting that Tregs actively proliferate *in vivo* [9]–[11], the percentage of the cells positive for MKI67 antigen identified by monoclonal antibody Ki-67 (MKI67; also known as Ki67) in Tregs of humans and humanized mice was significantly higher than those in Tms and Tns (Figure 1C and 1D). These results indicate that Tregs in humans and humanized mice are more actively cycling than Tns and Tms. Altogether, these results suggested that the profile and characteristics of CD4^+^ T cell subsets in humanized mice mirror those in healthy humans.

**Figure 2:**
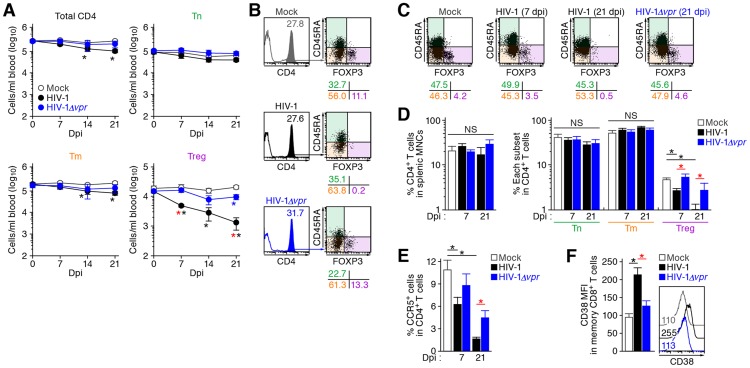
Dynamics of human CD4^+^ T cell subsets in humanized mice infected with R5 WT and *vpr*-deficient HIV-1. (A and B) Longitudinal analyses of the dynamics of human CD4^+^ T cell subsets in the PB of infected humanized mice. The numbers of total CD4^+^ T cells (CD45^+^ CD3^+^ CD4^+^ cells), Tns (CD45^+^ CD3^+^ CD4^+^ CD45RA^+^ FOXP3^−^ cells), Tms (CD45^+^ CD3^+^ CD4^+^ CD45RA^−^ FOXP3^−^ cells), and Tregs (CD45^+^ CD3^+^ CD4^+^ CD45RA^−^ FOXP3^+^ cells) in the PB of R5 WT HIV-1-infected mice (n = 8), R5 *vpr*-deficient HIV-1-infected mice (n = 8), and mock-infected mice (n = 12) were routinely quantified by flow cytometry and hematocytometry. Summarized results (A) and representative dot plots at 21 dpi (B) are shown, respectively. In panel B, the numbers in the histogram indicate the percentage of CD4^+^ cells in CD45^+^ cells, and the numbers under the dot plots indicate the percentage of the cells in each quadrant. (C and D) Cytopathic effect of WT and *vpr*-deficient HIV-1 in the spleen of humanized mice. The percentages of total CD4^+^ T cells, Tns, Tms, and Tregs in the splenic MNCs of WT HIV-1-infected mice (7 dpi, n = 19; 21 dpi, n = 8), *vpr*-deficient HIV-1-infected mice (7 dpi, n = 10; 21 dpi, n = 7), and mock-infected mice (n = 12) were routinely quantified by flow cytometry. Representative dot plots (C) and summarized results (D) are shown, respectively. In panel C, the numbers under the dot plots indicate the percentage of the cells in each quadrant. (E) The level of CCR5-expressing CD4^+^ T cells in infected humanized mice. The percentage of CCR5^+^ cells in the splenic CD4^+^ T cells of WT HIV-1-infected mice (7 dpi, n = 8; 21 dpi, n = 6), *vpr*-deficient HIV-1-infected mice (7 dpi, n = 8; 21 dpi, n = 6), and mock-infected mice (n = 8) was analyzed by flow cytometry. (F) The level of immune activation in infected humanized mice. The MFI of CD38 in memory CD8^+^ T cells (CD45^+^ CD3^+^ CD8^+^ CD45RA^−^ cells) in the spleen of WT HIV-1-infected mice (n = 5), *vpr*-deficient HIV-1-infected mice (n = 5), and mock-infected mice (n = 5) at 21 dpi was analyzed by flow cytometry. Representative histograms are shown on the right panel, and the numbers in the histogram indicate the MFI values. Statistical difference was determined by Welch's *t* test, and statistically significant differences (*P*<0.05) are shown as follows: mock versus WT HIV-1, black asterisk; mock versus HIV-1*Δvpr*, blue asterisk; and WT HIV-1 versus HIV-1*Δvpr*, red asterisk. NS, no statistical significance. Data represent mean ± SEM.

To investigate the dynamics of each CD4^+^ T cell subset after HIV-1 infection, 40 humanized mice were infected with a primary R5 HIV-1 isolate, strain JR-CSF [30]. As observed in HIV-1-infected individuals [15]–[17] and SIV-infected monkeys [18]–[20], we found that Tregs were preferentially and significantly decreased in the peripheral blood (PB) (Figure 2A and 2B) and the spleen (Figure 2C and 2D) of HIV-1-infected humanized mice until 21 days postinfection (dpi). However, because we have previously observed that surface CD4 molecules on HIV-1-infected cells in humanized mice are downregulated [21], [23], we evaluated whether this was the case in Tregs. Results showed that Tregs were positive for surface CD4 (i.e., CD4^−^ FOXP3^+^ cells were absent) (Figure S1), indicating that the disappearance of Tregs during the acute phase of infection was not due to surface CD4 down-regulation, but rather to depletion by HIV-1 infection. Since CCR5 is highly expressed on Tregs (Figure 1C and 1D), we further assessed the level of CCR5^+^ CD4^+^ T cells in R5 HIV-1-infected humanized mice. As shown in Figure 2E, we observed zthat the percentage of CCR5^+^ cells in the splenic CD4^+^ T cells of R5 HIV-1-infected mice was significantly lower than that of mock-infected mice (Figure 2E). These findings suggest that R5 HIV-1 infection induces severe depletion of CCR5^+^ CD4^+^ T cells including Tregs during acute infection.

It is well known that Tregs have the potential to suppress immune activation *in vivo*, and that the depletion of Tregs induces aberrant immune activation [7]. To address this possibility in HIV-1-infected humanized mice, we assessed the immune activation status at 21 dpi by staining with CD38, an activation marker [31], [32]. As shown in Figure 2F, the expression level of CD38 on memory CD8^+^ T cells in the spleen of HIV-1-infected mice was significantly higher than that of mock-infected mice. These results suggested that HIV-1 infection decreased Tregs in humanized mice and resulted in immune activation.

### Vpr depletes Tregs and enhances HIV-1 propagation in a coreceptor-dependent manner

**Figure 3:**
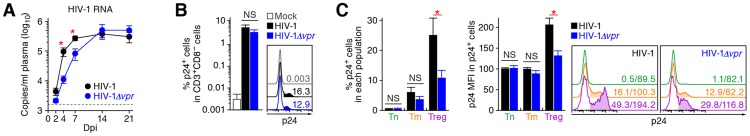
Dynamics of R5 WT and *vpr*-deficient HIV-1 infection in humanized mice. (A) Viral load in infected humanized mice. The amounts of viral RNA in the plasma of R5 WT HIV-1-infected mice (n = 30) and R5 *vpr*-deficient HIV-1-infected mice (n = 23) were routinely quantified. The horizontal broken line indicates the detection limit of the assay (1,600 copies/ml). (B and C) Infected cells in humanized mice. HIV-1-infected cells in the spleen of R5 WT HIV-1-infected mice (n = 19), R5 *vpr*-deficient HIV-1-infected mice (n = 10), and mock-infected mice (n = 10) at 7 dpi were analyzed by flow cytometry using an anti-HIV-1 p24 antibody. The percentages of p24^+^ cells in CD3^+^ CD8^−^ cells (B) and in each CD4^+^ T cell subset (C, left panel), and the MFI of p24 in p24^+^ cells of each CD4^+^ T cell subset (C, middle panel) are shown. Representative histograms are shown on the right panel. In panel B, the numbers in the histogram indicate the positivity. In panel C, the numbers in the histogram indicate the percentage of positive cells (left) and MFI values (right). Statistical difference was determined by Welch's *t* test, and statistically significant differences between WT HIV-1 versus HIV-1*Δvpr* (*P*<0.05) are shown with red asterisks. NS, no statistical significance. Data represent mean ± SEM.

As described in Introduction section, Vpr is pleiotropic and is known to induce cell cycle arrest at the G_2_ phase and apoptosis [1]. Since Tregs are highly proliferative *in vivo* (Figure 1), which is consistent with previous reports [9]–[11], we hypothesized that Tregs are highly susceptible to Vpr-mediated G_2_ arrest. To test this hypothesis, 32 humanized mice were infected with R5 *vpr*-deficient HIV-1 (HIV-1*Δvpr*; strain JR-CSF) [33]. Although the infectivities of R5 WT HIV-1 and R5 HIV-1*Δvpr* were comparable *in vitro* (Figure S2), the level of viral load in the plasma of HIV-1*Δvpr*-infected mice at 4 and 7 dpi was significantly lower than that of WT HIV-1-infected mice (Figure 3A). These results suggested that HIV-1*Δvpr* is less replicative than WT HIV-1 during initial stage of infection in humanized mice. We also investigated the dynamics of CD4^+^ T cells in HIV-1*Δvpr*-infected mice and found that the acute and severe depletion of Tregs after virus challenge was not observed in the PB (Figure 2A and 2B) and the spleen (Figure 2C and 2D). In addition, the level of CCR5^+^ CD4^+^ T cells in the spleen of HIV-1*Δvpr*-infected mice was significantly higher than that of WT HIV-1-infected mice (Figure 2E). Moreover, the immune activation, which was observed in WT HIV-1-infected mice, was not detected in HIV-1*Δvpr*-infected mice (Figure 2F). These findings suggested that Vpr enhances virus dissemination and induces Treg depletion leading to immune activation in humanized mice.

To address the association of Vpr with the rapid HIV-1 expansion *in vivo*, we next assessed the distribution of HIV-1-infected cells during acute infection (i.e., 7 dpi). As shown in Figure 3B, the percentage of the cells positive for p24, an HIV-1 antigen, in splenic CD3^+^ CD8^−^ cells of WT HIV-1-infected mice was comparable to that of HIV-1*Δvpr*-infected mice. We then examined the proportion of p24^+^ cells in each CD4^+^ T cell subset and found that Tregs were more positive for p24 than Tm and Tn in both WT HIV-1-infected and HIV-1*Δvpr*-infected mice (Figure 3C, left and right panels). In addition, we demonstrated that the percentage of p24^+^ Tregs in WT HIV-1-infected mice was significantly higher than that in HIV-1*Δvpr*-infected mice (Figure 3C, left and right panels). Moreover, in WT HIV-1 but not in HIV-1*Δvpr*-infected mice, the mean fluorescent intensity (MFI) of p24, which reflects the expression level of viral proteins in infected cells, was significantly higher in Tregs than in Tns and Tms (Figure 3C, middle and right panels). Taken together, these results suggested that Tregs were highly susceptible to HIV-1 infection and produced large amounts of the virus with Vpr responsible for augmenting this production.

**Figure 4:**
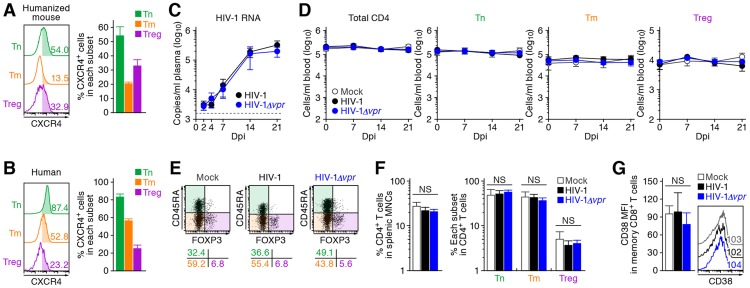
Dynamics of X4 WT and *vpr*-deficient HIV-1 infection in humanized mice. (A and B) CXCR4 expression on CD4^+^ T cell subsets in human and humanized mouse. Human CD4^+^ T cells isolated from the spleen of humanized mice (A, n = 8) and the PB of HIV seronegative humans (B, n = 6) were classified into Tn, Tm, and Treg as described in the legend of Figure 1. Representative dot plots and histograms are shown on the left, and the percentages of CXCR4^+^ cells in each subset are shown on the right. In the left panels, the numbers in each histogram indicate the positivity. (C) Viral load in infected humanized mice. The amounts of viral RNA in the plasma of X4 WT HIV-1-infected mice (n = 13) and X4 *vpr*-deficient HIV-1-infected mice (n = 11) were routinely quantified. The horizontal broken line indicates the detection limit of the assay (1,600 copies/ml). (D) Longitudinal analyses of the dynamics of human CD4^+^ T cell subsets in the PB of infected humanized mice. The numbers of total CD4^+^ T cells, Tns, Tms, and Tregs in the PB of WT HIV-1-infected mice (n = 9), *vpr*-deficient HIV-1-infected mice (n = 9), and mock-infected mice (n = 8) were routinely quantified by flow cytometry and hematocytometry. (E and F) Cytopathic effect of WT and *vpr*-deficient HIV-1 in the spleen of humanized mice. The percentages of total CD4^+^ T cells, Tns, Tms, and Tregs in the splenic MNCs of WT HIV-1-infected mice (n = 8), *vpr*-deficient HIV-1-infected mice (n = 8), and mock-infected mice (n = 8) at 21 dpi were routinely quantified by flow cytometry. Representative dot plots (E) and summarized results (F) are shown, respectively. In panel E, the numbers on the right of the dot plots indicate the percentage of the cells in each quadrant. (G) The level of immune activation in infected humanized mice. The MFI of CD38 in memory CD8^+^ T cells in the spleen of WT HIV-1-infected mice (n = 5), *vpr*-deficient HIV-1-infected mice (n = 5), and mock-infected mice (n = 5) at 21 dpi was analyzed by flow cytometry. Representative histograms are shown on the right panel, and the numbers in the histogram indicate the MFI values. NS, no statistical significance. Data represent mean ± SEM.

These findings raised the possibility that the preferential HIV-1 infection in Tregs was due to their high CCR5 expression (Figure 1C and 1D). To demonstrate this possibility, we assessed the expression level of CXCR4, another coreceptor for HIV-1, in each CD4^+^ T cell subset. In both humans and humanized mice, we found that CXCR4 was broadly expressed in all CD4^+^ T cell subsets and was highly expressed on Tns than Tms and Tregs (Figure 4A and 4B). Then, 13 humanized mice were infected with an X4 WT HIV-1 (strain NL4-3) [34], while 11 humanized mice were infected with an X4 HIV-1*Δvpr* (strain NL4-3) [2]. The infectivities of X4 WT HIV-1 and X4 HIV-1*Δvpr* were comparable *in vitro* (Figure S3). In contrast to the observations in R5 HIV-1-infected humanized mice (Figure 3A), the viral load of X4 WT HIV-1 and was comparable to that of X4 *vpr*-deficient HIV-1 (Figure 4C). In addition, the depletion of Tregs during the acute phase of infection, which was found in R5 HIV-1-infected mice (Figure 2A–2D), was not observed in the PB (Figure 4D) and the spleen (Figure 4E and 4F) of X4 WT HIV-1-infected mice. Furthermore, we did not observe the immune activation in X4 HIV-1-infected mice during acute infection (Figure 4G). Taken together, these findings strongly suggest that the preferential HIV-1 infection and the Treg depletion leading to immune activation during acute infection are dependent on the coreceptor usage of HIV-1.

### Vpr induces a significant level of G_2_ cell cycle arrest in infected Tregs

**Figure 5:**
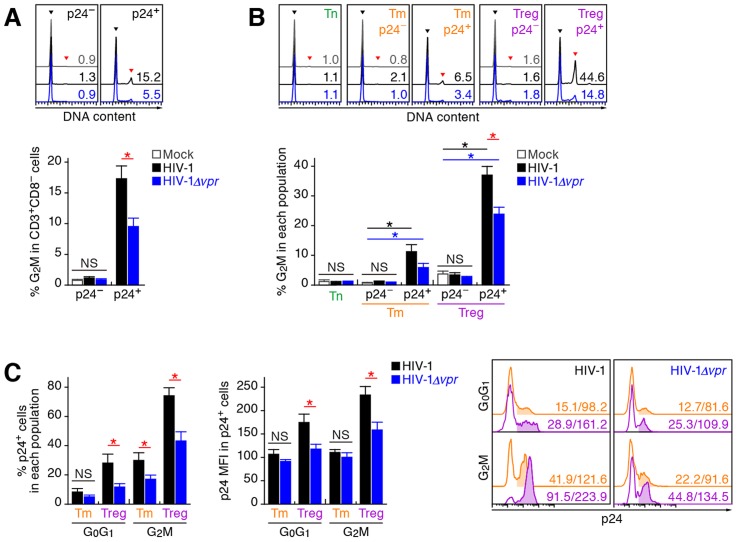
Effect of Vpr on G_2_ cell cycle arrest in infected humanized mice. Splenic MNCs of WT HIV-1-infected mice (n = 12), *vpr*-deficient HIV-1-infected mice (n = 11), and mock-infected mice (n = 15) at 7 dpi were analyzed by flow cytometry using Hoechst33342 and an anti-HIV-1 p24 antibody. (A and B) The percentages of G_2_M cells in CD3^+^ CD8^−^ cells (A) and in each population (B) are shown, respectively. Representative histograms are shown on the right panel. The black arrowhead indicates the peak of G_0_G_1_ cells, and the red arrowhead indicates the peak of G_2_M cells. The numbers in the histogram indicate the percentage of G_2_M cells in each population. (C) The percentage of p24^+^ cells in each population (left) and the MFI of p24 in p24^+^ cells of each population (middle). Representative histograms are respectively shown. The numbers in the histogram indicate the percentage of positive cells (left) and MFI values (right). Statistical differences were determined by Welch's *t* test, and statistically significant differences (*P*<0.05) are shown as follows: mock versus WT HIV-1, black asterisk; mock versus HIV-1*Δvpr*, blue asterisk; and WT HIV-1 versus HIV-1*Δvpr*, red asterisk. NS, no statistical significance. Data represent mean ± SEM.

Extensive *in vitro* studies have reported that Vpr can cause cell cycle arrest at the G_2_ phase [1]. To investigate the cell cycle condition of R5 HIV-1-infected cells in humanized mice at 7 dpi, cellular DNA content was quantified by Hoechst staining. Although the percentages of p24-negative cells at the G_2_M phase in the spleen of WT HIV-1-infected and HIV-1*Δvpr*-infected cells were similar to those of mock-infected mice, a significant level of p24-positive cells at the G_2_M phase in both WT HIV-1-infected and HIV-1*Δvpr*-infected mice were detected (Figure 5A). Moreover, we found that the percentage of p24^+^ cells at the G_2_M phase in WT HIV-1-infected mice was significantly higher than that in HIV-1*Δvpr*-infected mice (Figure 5A), suggesting that Vpr expressed in infected cells induced G_2_ cell cycle arrest *in vivo*.

We next analyzed the level of G_2_ arrest in each CD4^+^ T cell subset. Since p24^+^ cells were faintly detected in the Tn subset (Figure 3C; 0.33±0.1% for WT HIV-1, 0.35±0.1% for HIV-1*Δvpr*), we focused on Tms and Tregs. In both subsets, the percentages of G_2_M cells in p24^−^ cells of WT HIV-1-infected and HIV-1*Δvpr*-infected mice were similar to those of mock-infected mice (Figure 5B). In contrast, we detected a significant level of p24^+^ cells at the G_2_M phase in Tms and Tregs (Figure 5B). Of note, the percentage of G_2_M cells in p24^+^ Tregs of WT HIV-1-infected mice reached a maximum of 37.1±2.8% and was significantly higher than that of HIV-1*Δvpr*-infected mice (Figure 5B). These results suggested that the level of Vpr-mediated G_2_ arrest was the highest in HIV-1-infected Tregs.

Since it has been suggested that the G_2_ arrest in HIV-1-infected cells results in the augmentation of virus production [3], [35], we next focused on the relationship between the HIV-1 production potential and cell cycle condition in Tms and Tregs. Figure 5C illustrated that G_2_M cells displayed higher percentages of p24-positive cells than G_0_G_1_ cells in both Tm and Treg. Surprisingly, 74.1±5.4% of Tregs at the G_2_M phase in WT HIV-1-infected mice were positive for p24 (Figure 5C, left and right panels), and the p24 MFI in p24^+^ Tregs at G_2_M phase was highest (Figure 5C, middle and right panels). Taken together, these findings suggested that the majority of Tregs were infected with HIV-1 and arrested at the G_2_ phase by Vpr, resulting in the augmentation of HIV-1 production during acute infection.

### Vpr directly induces apoptosis in infected Tregs associated with G_2_ cell cycle arrest

**Figure 6:**
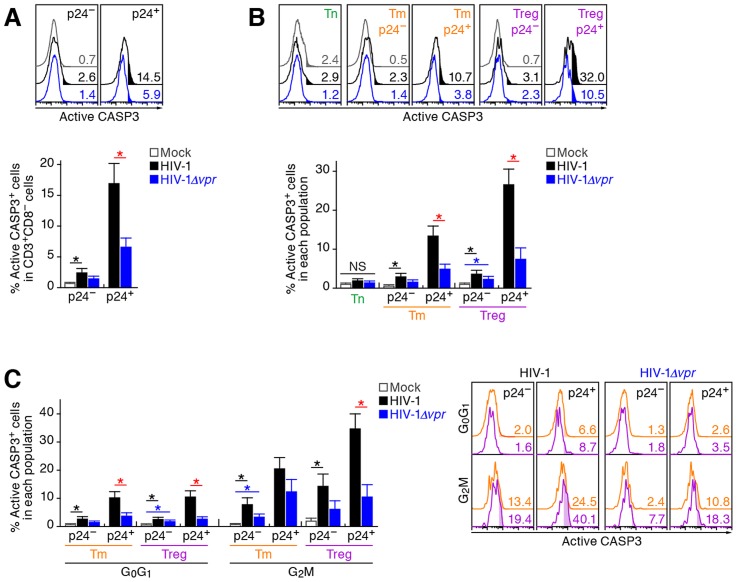
Effect of Vpr on apoptosis and its relevance in G_2_ cell cycle arrest in infected humanized mice. Splenic MNCs of WT HIV-1-infected mice (n = 7), *vpr*-deficient HIV-1-infected mice (n = 7), and mock-infected mice (n = 9) at 7 dpi were analyzed by flow cytometry using anti-active CASP3 and anti-HIV-1 p24 antibodies without (A and B) or with (C) or Hoechst33342. (A and B) Effect of Vpr on apoptosis. The percentages of active CASP3^+^ cells in CD3^+^ CD8^−^ cells (A) and in each population (B) are shown, respectively. Representative histograms are shown on the right panel. The numbers in the histogram indicate the percentage of active CASP3^+^ cells in each population. (C) Relevance between G_2_ arrest and apoptosis. The percentage of active CASP3^+^ cells in each population is shown. Representative histograms are respectively shown. The numbers in the histogram indicate the percentage of active CASP3^+^ cells in each population. Statistical differences were determined by Welch's *t* test, and statistically significant differences (*P*<0.05) are shown as follows: mock versus WT HIV-1, black asterisk; mock versus HIV-1*Δvpr*, blue asterisk; and WT HIV-1 versus HIV-1*Δvpr*, red asterisk. NS, no statistical significance. Data represent mean ± SEM.

In addition to the augmentation of viral replication by Vpr, we also observed a severe depletion of Tregs in R5 WT HIV-1-infected humanized mice (Figure 2A–2D). It is known that Vpr can induce apoptosis through a caspase 3/8 (CASP3/8)-dependent pathway [1]. Therefore, we next analyzed the level of active CASP3, which is a direct inducer of apoptosis, in infected humanized mice. In the population of p24-negative cells, we found a significant increase of active CASP3^+^ cells in WT HIV-1-infected mice (Figure 6A). Additionally, in both WT HIV-1-infected and HIV-1*Δvpr*-infected mice, the percentage of active CASP3 in p24^+^ cells was significantly higher than that in p24^−^ cells, yet the percentage of active CASP3 in p24^+^ cells of WT HIV-1-infected cells was significantly higher than that of HIV-1*Δvpr*-infected mice (Figure 6A).

We then evaluated the magnitude of apoptosis in each CD4^+^ T cell subset. As shown in Figure 6B, the percentage of active CASP3^+^ cells in p24^−^ Tms and Tregs of WT HIV-1-infected mice significantly increased when compared with those of mock-infected mice. On the other hand, the percentage of active CASP3^+^ cells was significantly increased in p24^+^ cells and was highest in p24^+^ Tregs of WT HIV-1-infected mice (26.6±3.9%; Figure 6B), suggesting that Tregs are highly sensitive to Vpr-mediated apoptosis.

In addition to the apoptosis directly induced by Vpr, accumulating evidence has suggested a role for innate immune activation, including NK cells, in the CD4^+^ T cell depletion after primary HIV-1 infection in individuals [36], [37]. Also, it has been recently reported that Vpr upregulates the surface expression of some NK receptor ligands, such as UL16 binding protein 2 (ULBP2), which leads to NK cell-dependent cell death [38], [39]. These reports led to the hypothesis that Vpr upregulates the expression level of ULBP2 on HIV-1-infected Tregs and enhances NK cell-dependent cell death. To address this possibility, we assessed the expression level of ULBP2 in infected humanized mice. However, the expression level of ULBP2 on the surface of WT HIV-1-infected cells was comparable to those of HIV-1*Δvpr*-infected cells, uninfected cells, and the CD4^+^ T cells in mock-infected mice (Figure S4). Taken together, these results suggested that the decrease of Tregs in R5 WT HIV-1-infected mice was not dependent on the NK cell-dependent cell death but due to Vpr expressed in infected cells.

In order to investigate the relationship between G_2_ cell cycle arrest and apoptosis, both of which are mediated by Vpr, we performed p24 staining in combination with Hoechst and active CASP3 staining. In each CD4^+^ T cell subset positive for p24, the percentage of active CASP3^+^ cells at G_2_M was significantly higher than that at the G_0_G_1_ phase (Figure 6C). Moreover, the percentage of active CASP3^+^ cells was highest in p24^+^ Tregs at G_2_M in WT HIV-1-infected mice (35.9±5.4%; Figure 6C), strongly suggesting that Vpr-mediated apoptosis was most efficiently induced in infected Tregs arrested at the G_2_ phase.

### Treg depletion can trigger immune activation and augmented HIV-1 propagation *in vivo*

**Figure 7:**
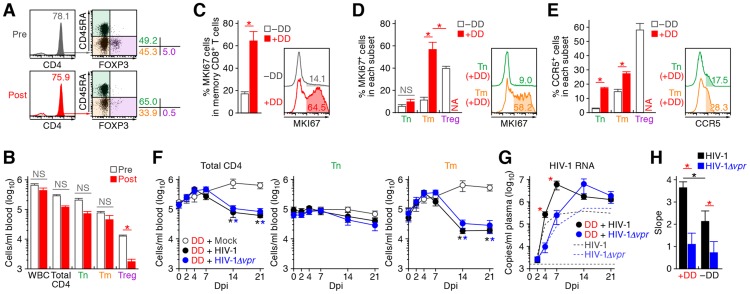
Augmentation of Vpr's effect and HIV-1 propagation by Treg depletion. (A to D) Evaluation of Treg depletion by treatment with DD. DD was administrated into humanized mice (n = 14) as described in Materials and Methods. (A and B) Specific depletion of Tregs by treatment with DD. The levels of human white blood cells (WBC; CD45^+^ cells) and CD4^+^ T cell subsets in PB of humanized mice before and after the DD treatment for 3 days were compared. Representatives (A) and the numbers of each human leukocytes in PB (B) are shown. In panel A, the numbers in the histogram indicate the percentage of CD4^+^ cells in CD45^+^ CD3^+^ cells, and the numbers on the right of the dot plots indicate the percentage of the cells in each quadrant. (C and D) Immune activation by treatment with DD. The percentages of MKI67^+^ cells in memory CD8^+^ T cells (C) and in each CD4^+^ T cell subset (D) in the spleen of humanized mice treated with (n = 5) or without (n = 8) DD for 7 days are shown, respectively. (E) Up-regulation of CCR5 expression by DD treatment. The percentage of CCR5^+^ cells in each CD4^+^ T cell subset in the spleen of humanized mice treated with (n = 5) or without (n = 8) DD for 7 days is shown. In panels C to E, the numbers in the histogram indicate positivity. (F to H) Dynamics of HIV-1 infection in DD-treated humanized mice. (F) The numbers of peripheral CD4^+^ T cells, Tns, Tms, and Tregs (F) and the amounts of viral RNA in the plasma (G) of R5 WT HIV-1-infected DD-treated mice (n = 13), R5 *vpr*-deficient HIV-1-infected DD-treated mice (n = 13), and mock-infected DD-treated mice (n = 8) were routinely quantified as described in the legends of Figure 2A and 3A, respectively. In panel G, the broken black and blue lines indicate the averages of WT HIV-1-infected mice (n = 30) and *vpr*-deficient HIV-1-infected mice (n = 23) without DD treatment, which corresponds to the results shown in Figure 3A. The horizontal broken line indicates the detection limit of the assay (1,600 copies/ml). (H) Kinetics of viral expansion. The slopes of the amounts of viral RNA in the plasma of WT HIV-1-infected DD-treated mice (n = 13), *vpr*-deficient HIV-1-infected DD-treated mice (n = 13), WT HIV-1-infected mice (n = 30) and *vpr*-deficient HIV-1-infected mice (n = 23) until 7 dpi are shown. Statistical difference was determined by Welch's *t* test. In panels B to E, statistically significant differences (*P*<0.05) are indicated by red asterisks. In panels F and G, statistically significant differences (*P*<0.05) are shown as follows: mock versus WT HIV-1, black asterisk; mock versus HIV-1*Δvpr*, blue asterisk; and WT HIV-1 versus HIV-1*Δvpr*, red asterisk. In panel H, statistically significant differences (*P*<0.05) are shown as follows: with and without DD treatment, black asterisk; and WT HIV-1 versus HIV-1*Δvpr*, red asterisk. NS, no statistical significance. Data represent mean ± SEM. NA, not analyzed.

The aforementioned findings suggested that Vpr promotes R5 HIV-1 propagation during the acute phase of infection by exploiting proliferating CCR5^+^ CD4^+^ T cells including Tregs *in vivo*. In addition, Vpr is associated with the rapid decrease of Tregs, leading to immune activation. Since it is known that HIV-1 replicates more efficiently in activated CD4^+^ T cells than non-activated CD4^+^ T cells [40], [41], our findings suggested that the immune activation induced by Vpr-mediated Treg depletion led to the augmented viral propagation *in vivo*. To address this possibility, denileukin diftitox (DD), which is known to specifically target and deplete Tregs, was intraperitoneally treated into humanized mice. As shown in Figure 7A and 7B, Tregs were specifically and significantly depleted by treatment with DD for 3 days, while the cell numbers of the other populations such as CD45^+^ human white blood cells, total CD4^+^ T cells, Tns, and Tms did not change significantly. We also found that the Treg depletion by DD induced immune activation and proliferation of splenic memory CD8^+^ T cells (Figure 7C). Interestingly, the percentage of MKI67^+^ cells in the Tms of DD-treated humanized mice was significantly higher than those in Tms and Tregs of untreated humanized mice (Figure 7D). In addition, the levels of CCR5 on Tms and Tns in DD-treated mice were significantly higher than that in untreated mice (Figure 7E), suggesting that the population size of proliferating CCR5^+^ CD4^+^ T cells in DD-treated humanized mice is greater than that in untreated humanized mice.

R5 WT and *vpr*-deficient HIV-1 (strain JR-CSF) were then inoculated into 13 DD-treated humanized mice, respectively. As shown in Figure 7F, the number of CD4^+^ T cells, particularly Tms, in the PB of DD-treated uninfected mice gradually increased, while that those of DD-treated WT and *vpr*-deficient HIV-1 infected mice severely decreased after 7 dpi. We also observed a gradual increase of memory CD8^+^ T cells in the PB of DD-treated humanized mice regardless of HIV-1 infection (Figure S5). It was of particular importance that rapid and massive HIV-1 replication in DD-treated mice compared with untreated mice infected with either virus, and that the viral load in DD-treated WT HIV-1-infected mice was significantly higher than that in DD-treated HIV-1*Δvpr*-infected mice at 4 and 7 dpi (Figure 7G). Furthermore, the slope of virus growth in DD-treated WT HIV-1-infected mice was significantly higher than those of DD-treated HIV-1*Δvpr*-infected mice and untreated WT HIV-1-infected mice (Figure 7H). Taken together, these findings suggest that R5 HIV-1 massively propagates under an activated condition, and that Vpr enhances viral expansion in CCR5^+^ proliferating CD4^+^ T cell population.

## Discussion

The fact that *vpr* is conserved in transmitted/founder viruses in infected individuals [42] may indicate its importance during the acute phase of HIV-1 propagation. However, even though there is abundant evidence of Vpr's roles in G_2_ arrest and apoptosis *in vitro* [1], [43], [44], its impact on for HIV-1 replication *in vivo* remains unclear. In this study, we demonstrated that Vpr augments R5 HIV-1 propagation by exploiting proliferating CCR5^+^ CD4^+^ T cells including Tregs during acute infection. We also observed significant levels of Vpr-dependent G_2_ arrest and apoptosis in R5 HIV-1-infected Tregs, which may result in the Treg depletion and subsequent immune activation. This is the first report to directly demonstrate that Vpr positively affects HIV-1 replication by taking advantage of Tregs *in vivo*.

A previous study has demonstrated that Tregs highly express CCR5, correlating with their high susceptibility to R5 HIV-1 *in vitro* [15]. Here, by using a humanized mouse model, we demonstrated that Tregs express higher level of CCR5 (Figure 1) and are highly susceptible to R5 HIV-1 infection *in vivo* (Figure 3). In addition, it is well known that HIV-1 replicates more efficiently in activated/proliferating cells than in non-activated cells [40], [41]. Consistent with previous reports [9]–[11], we showed that Tregs are highly proliferative *in vivo* when compared with the other CD4^+^ T cell subsets such as Tns and Tms (Figure 1). Therefore, it is reasonable to assume that R5 HIV-1 efficiently replicates in Tregs of humanized mice because of their higher CCR5 expression level and higher proliferating status. Moreover, in line with the previous observations that Vpr arrests the cell cycle of HIV-1-infected cells at G_2_ phase where LTR-driven HIV-1 transcription is most active [3], [35], we found that the MFI of p24, which reflects the expression level of viral proteins, in Tregs of WT HIV-1-infected mice was ∼2-fold higher than that of HIV-1*Δvpr*-infected mice, while expression levels in Tns and Tms were comparable between WT and *vpr*-deficient HIV-1 (Figure 3C). Furthermore, we revealed that Vpr-dependent G_2_ cell cycle arrest was efficiently occurred in infected Tregs (Figure 5B), and that both the percentage p24^+^ cells and the p24 MFI was highest in WT HIV-1-infected Tregs at G_2_ phase (Figure 5C). Taken together, these findings strongly suggest that Vpr promotes R5 HIV-1 replication during acute infection by increasing the viral production in Tregs.

In contrast to the findings in R5 HIV-1-infected humanized mouse model, we observed neither the acceleration of virus replication by Vpr during the acute phase of HIV-1 infection (Figure 4C), nor the Treg depletion (Figure 4D–4F), nor subsequent immune activation (Figure 4G) in X4 HIV-1-infected humanized mice. In Tregs, CCR5 is predominantly expressed (Figure 1C and 1D), whereas CXCR4 is broadly expressed in all CD4^+^ T cell subsets (Figure 4A and 4B), which is consistent with previous findings [15], [28]. Therefore, these results suggest that the Vpr-dependent augmentation of HIV-1 replication during acute infection is dependent on viral tropism and is restricted to R5 HIV-1. Regarding HIV-1 tropism, it is of particular importance that R5 HIV-1 is the major clinical isolates from patients, along with transmitted/founder viruses [42], [45], [46], while X4 HIV-1 occasionally emerges during the onset of AIDS [47], [48]. Therefore, the findings in R5 HIV-1-infected humanized mice more properly reflect those in patients than those in X4 HIV-1-infected mice, and the role of Vpr in R5 HIV-1-infected humanized mice is physiologically more relevant.

The concept that Vpr augments R5 HIV-1 replication by utilizing proliferating CCR5^+^ CD4^+^ T cells is further supported by the DD treatment experiments (Figure 7): the human leukocytes including Tms in the mice treated with DD were highly proliferative and the Tms in DD-treated mice expressed higher level of CCR5. Moreover, R5 HIV-1 propagated more efficiently when compared with the untreated mice. Interestingly, it has been reported that Vpr enhances HIV-1 LTR-driven transcription in cycling CD4^+^ T cells but not in non-cycling cells [3]. Taken together, these findings suggest that Vpr-dependent promotion of R5 HIV-1 production during acute infection is attributed to the target cell tropism of HIV-1 and the activated/proliferative status of the target cells.

There is a longstanding dogma that the immune activation caused by HIV-1/SIV infection closely associates with the disease progression [49]. Regarding the triggering of immune activation, previous studies have suggested that the immune activation in HIV-1-infected individuals and SIV-infected monkeys can be caused by (1) massive infection and loss of CD4^+^ T cells [50], [51]; (2) inflammatory cytokines [52], [53]; and (3) microbial translocation from the luminal intestinal tract [54]. In this study, Treg depletion and immune activation were observed in R5 but not X4 HIV-1-infected humanized mice (Figure 2 and 4). These findings are consistent with previous observations in HIV-1-infected patients [15]–[17], SIV-infected monkeys [18]–[20], and a CCR5/CXCR4 dual-tropic HIV-1-infected humanized mouse model [28]. Particularly noteworthy is that *vpr*-deficient HIV-1-infected humanized mice showed neither Treg depletion nor immune activation. These findings raise a possibility that Vpr is associated with the induction of immune activation by depleting Tregs. Since the physiological role of Tregs *in vivo* is to suppress excessive immune activation [7], it is conceivable that Vpr-mediated Treg depletion can be one of the triggers for immune activation in HIV-1-infected individuals. However, the mechanism of the immune activation by HIV-1/SIV infection still remains unsolved for more than two decades of intense research, and there are lines of other possibilities such as the activation of dendritic cells/macrophages due to higher number of cell death [55]–[57] and the actual depletion of myeloid-derived suppressor cells [58], [59] by direct or indirect virus infection. Although our results suggest that Vpr is associated with the acute Treg depletion and subsequent immune activation in R5 HIV-1-infected humanized mice, further investigations is necessary to elucidate the mechanisms of the immune activation by HIV-1/SIV infection.

In DD-treated humanized mice, we observed the activation/proliferation (Figure 7C) and the expansion (Figure S5) of memory CD8^+^ T cells. As a previous report using R5 HIV-1-infected humanized mice showed that the depletion of CD8^+^ T cells accelerates HIV-1 replication [60], these findings raise a possibility that the expanded memory CD8^+^ T cells restrict HIV-1 replication in DD-treated humanized mice. However, the previous study [60] depleted CD8^+^ T cells in R5 HIV-1-infected humanized mice during chronic infection (i.e, 5–7 weeks postinfection) and observed the increase of virus growth 2 weeks after CD8^+^ T cell depletion. On the other hand, although the increase of memory CD8^+^ T cells was observed in DD-treated humanized mice after 4 or 7 dpi (Figure S5), here we particularly focused on the dynamics of HIV-1 infection during the acute phase (i.e., until 7 dpi) and observed a sharp increase of HIV-1 replication in DD-treated mice prior to the expansion of memory CD8^+^ T cells (Figure 7G). Moreover, the activation and expansion of CD8^+^ T cells were detected in DD-treated humanized mice regardless of HIV-1 infection, strongly suggesting that this CD8^+^ T cell expansion is not triggered by HIV-1 infection but by the DD-mediated Treg depletion. Furthermore, although the expansion of memory CD8^+^ T cells during chronic infection has been observed in certain HIV-1-infected human HSC-transplanted humanized mouse models [61]–[63] including ours [25], it is controversial whether or not the human CD8^+^ T cells differentiated in human HSC-transplanted humanized mouse models possess the potential to efficiently elicit acquired immune responses against pathogens including HIV-1 [64]–[66]. These findings suggest that the expanded CD8^+^ T cells in DD-treated humanized mice have smaller effect on the virus growth during the acute phase of HIV-1 infection.

Soluble Vpr proteins are secreted from infected cells and can be detected in patient sera [67], [68]. In p24-negative cells of WT HIV-1-infected mice, we found a significant level of apoptosis (Figure 6A and 6B), while G_2_ arrest was not observed (Figure 5A and 5B). These results suggest that soluble Vpr can trigger apoptosis but not G_2_ arrest in bystander cells. In fact, it was reported that the Vpr expressed in HIV-1-infected cells robustly induce both G_2_ arrest and apoptosis, while soluble Vpr secreted from HIV-1-infected cells can induce apoptosis but not G_2_ arrest [69]. However, in addition to WT HIV-1-infected cells, G_2_ arrest was also partially observed in HIV-1*Δvpr*-infected cells (Figure 5A and 5B). In this regard, it has been reported that another accessory protein of HIV-1, Vif, is also able to cause G_2_ arrest in a Vpr-independent manner [70]–[72], strongly suggesting that the G_2_ arrest in HIV-1*Δvpr*-infected cells is induced by Vif. Although the significance of functional redundancy of Vpr and Vif for G_2_ arrest remains unclear, further studies using humanized mice will reveal their impact.

In summary, we demonstrated for the first time that one of the major roles of Vpr in HIV-1 infection and pathogenesis is to enhance R5 HIV-1 propagation by exploiting proliferating CCR5^+^ CD4^+^ T cells including Tregs during acute infection, which can subsequently induce immune activation. Our findings suggest that the action of Vpr *in vivo* may provide HIV-1 with an optical condition to replicate and facilitate HIV-1 expansion *in vivo*.

## Materials and Methods

### Ethics statement

All procedures including animal studies were conducted following the guidelines for the Care and Use of Laboratory Animals of the Ministry of Education, Culture, Sports, Science and Technology, Japan. These studies were approved by the Institutional Animal Care and Use Committees (IACUC)/ethics committee of Kyoto University (protocol number D13–25). All protocols involving human subjects were reviewed and approved by the Kyoto University institutional review board. Informed written consent from human subjects was obtained in this study.

### Humanized mice

NOD.Cg-*Prkdc^scid^ Il2rg^tm1Sug^/Jic* (NOD/SCID *Il2rg^−/−^*) mice [73] were obtained from the Central Institute for Experimental Animals (Kawasaki, Kanagawa, Japan). The mice were maintained under specific-pathogen-free conditions and were handled in accordance with the regulations of the IACUC/ethics committee of Kyoto University. Human CD34^+^ HSCs were isolated from human fetal liver as described previously [74]. The humanized mouse (NOG-hCD34 mouse) was constructed as previously described [21]–[24]. Briefly, 164 newborn (aged 0 to 2 days) NOG mice from 38 litters were irradiated with X-ray (10 cGy per mouse) by an RX-650 X-ray cabinet system (Faxitron X-ray Corporation) and were then intrahepatically injected with the obtained human fetal liver-derived CD34^+^ cells (7.5×10^4^ to 25×10^4^ cells). A list of the humanized mice used in this study is summarized in Table S1.

### Virus preparation and infection

Virus solutions of R5 WT HIV-1_JR-CSF_ [30], R5 *vpr*-deficient HIV-1_JR-CSF_ [33], X4 WT HIV-1_NL4-3_ [34], and X4 *vpr*-deficient HIV-1_NL4-3_ [2] were prepared and titrated as previously described [23]. Virus solutions of 10^5^ 50% tissue culture infectious doses (TCID_50_) were intraperitoneally inoculated into NOG-hCD34 mice. RPMI 1640 was used for mock infection.

### HIV-1 RNA quantification, TZM-bl assay, and western blotting

The amount of HIV-1 RNA in plasma was quantified by Bio Medical Laboratories, Inc. TZM-bl assay and Western blotting were performed as previously described [22], [23]. For Western blotting, mouse anti-Vpr antibody (clone 8D1) [68] and goat anti-p24 antiserum (ViroStat) were used.

### PB collection and isolation of splenic mononuclear cells

PB and plasma were routinely collected as previously described [21]–[24]. Splenic human mononuclear cells (MNCs) were isolated as previously described [22]–[24].

### Flow cytometry and hematocytometry

Flow cytometry was performed with FACSCanto (BD Biosceiences) as previously described [21]–[24]. Hematocytometry was performed with Celltac alpha MEK-6450 (Nihon Kohden Co) as previously described [23], [24]. Briefly, 10 µl of the PB of humanized mice were used for hematometry, and the number of MNCs per microliter was measured. The antibodies used in flow cytometry analysis are listed in Table S2. For cell cycle analysis, cellular DNA was stained with Hoechst33342 (Invitrogen) as previously described [21], and DNA contents were analyzed by using ModFit LT software (Verify software house) according to the manufacture's protocol and as previously reported [72]. For the measurement of the level of apoptosis, anti-active CASP3 antibody conjugated with PE (BD Biosciences; Table S2) was used according to the manufacture's procedure.

### Denileukin diftitox treatment for Treg depletion

Denileukin diftitox (DD; IL-2 conjugated with diphtheria toxin) were purchased from Ligand Pharma, Co. For Treg depletion in humanized mice, DD (400 µg/200 µl in PBS) were intraperitoneally treated once per day. For HIV-1 infection following DD treatment, the humanized mice treated with DD for 3 days were intraperitoneally inoculated with virus solutions of 10^5^ TCID_50_. RPMI 1640 was used as the mock infection. To maintain Treg depletion following virus inoculation, DD was intraperitoneally treated once per day.

### Statistical analyses

Data were expressed as averages with SEMs. Significant differences (*P*<0.05) were determined by Welch's *t* test or Student's *t* test.

### Accession numbers

SwissProt (http://www.uniprot.org/) or GenBank (http://www.ncbi.nlm.nih.gov/genbank) accession numbers for the proteins mentioned in the text are as follows: CD3 (P07766); CD4 (P01730); CD8 (NP_001759.3); CD25 (NP_000408.1); CD38 (P28907); CD45 (NP_002829.3); CD45RA (P08575); CD127 (P16871); CASP3 (P42574); CCR5 (P51681); CXCR4 (P61073); CTLA4 (P16410); FOXP3 (Q9BZS1); MKI67 (P46013); ULBP2 (Q9BZM5). These proteins were detected by flow cytometry using the antibodies listed in Table S2. The accession numbers from GenBank (http://www.ncbi.nlm.nih.gov/genbank) for the viruses mentioned in the text are as follows: HIV-1 strain JR-CSF (M38429.1); HIV-1 strain NL4-3 (M19921.2).

## Supporting Information

Figure S1 **Depletion of Treg by WT HIV-1 infection.** The percentage of FOXP3^+^ CD4^−^ cells in splenic MNCs of WT HIV-1-infected mice (n = 5) and mock-infected mice (n = 5) at 21 dpi are shown. Representative dot plots are shown below. The numbers under the dot plots correspond to the percentage in each quadrant. NS, no statistical significance.
(TIF)

Figure S2 **Infectivity of R5 WT and** ***vpr*** **-deficient HIV-1.** R5 WT and *vpr*-deficient HIV-1 (strain JR-CSF) were prepared as described in Materials and Methods. (Top) Western blot analyses of the virions. (Bottom) TZM-bl assay. Prepared virus solutions were inoculated into TZM-bl indicator cells. The infectivities of these viruses were quantified as described in Materials and Methods and were normalized to the amount of p24. The assay was performed in triplicate. NS, no statistical significance.
(TIF)

Figure S3 **Infectivity of X4 WT and** ***vpr*** **-deficient HIV-1.** X4 WT and *vpr*-deficient HIV-1 (strain NL4-3) were prepared as described in Materials and Methods. (Top) Western blot analyses of the virions. (Bottom) TZM-bl assay. Prepared virus solutions were inoculated into TZM-bl indicator cells. The infectivities of these viruses were quantified as described in Materials and Methods and were normalized to the amount of p24. The assay was performed in triplicate. NS, no statistical significance.
(TIF)

Figure S4 **No association of ULBP2 with the Treg depletion observed in WT HIV-1-infected mice.** Splenic MNCs of WT HIV-1-infected mice (n = 7), *vpr*-deficient HIV-1-infected mice (n = 7), and mock-infected mice (n = 7) at 7 dpi were analyzed by flow cytometry using an anti-ULBP2 and an anti-HIV-1 p24 antibodies. The percentages of ULBP2^+^ cells in CD3^+^ CD8^−^ cells (A) and in each population (B) are respectively shown. Representative histograms are shown on the right. The numbers in histogram indicate the percentage of active CASP3^+^ cells in each population. Statistical difference was determined by Welch's *t* test. NS, no statistical significance.
(TIF)

Figure S5 **Expansion of memory CD8^+^ T cells in DD-treated humanized mice.** The numbers of total CD8^+^ T cells (CD45^+^ CD3^+^ CD8^+^ cells), naïve CD8^+^ T cells (CD45^+^ CD3^+^ CD8^+^ CD45RA^+^ cells), and memory CD8^+^ T cells (CD45^+^ CD3^+^ CD8^+^ CD45RA^−^ cells) in the PB of R5 WT HIV-1-infected DD-treated mice (n = 13), R5 *vpr*-deficient HIV-1-infected DD-treated mice (n = 13), and mock-infected DD-treated mice (n = 8) were routinely quantified by flow cytometry and hematocytometry.
(TIF)

Table S1 **Humanized mice used in this study.** A full list of the 132 humanized mice used in this study.
(PDF)

Table S2 **Antibodies used in flow cytometry analyses.** A full list of antibodies used in this study.
(PDF)
