# Supplementary figures and images for: HIV-1 Vpr Accelerates Viral Replication during Acute Infection by Exploitation of Proliferating CD4+ T Cells In Vivo
Source: PLoS Pathog. 2013 Dec 5;9(12):e1003812. doi: 10.1371/journal.ppat.1003812 (PMC3855622; doi:10.1371/journal.ppat.1003812)

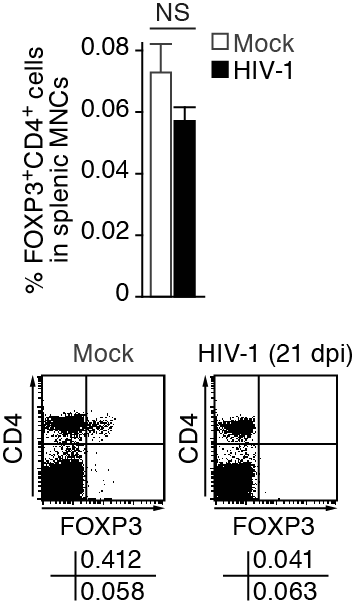

Supplement: Figure S1 — Depletion of Treg by WT HIV-1 infection. The percentage of FOXP3+ CD4− cells in splenic MNCs of WT HIV-1-infected mice (n = 5) and mock-infected mice (n = 5) at 21 dpi are shown. Representative dot plots are shown below. The numbers under the dot plots correspond to the percentage in each quadrant. NS, no statistical significance. (TIF) [file ppat.1003812.s001.tif]

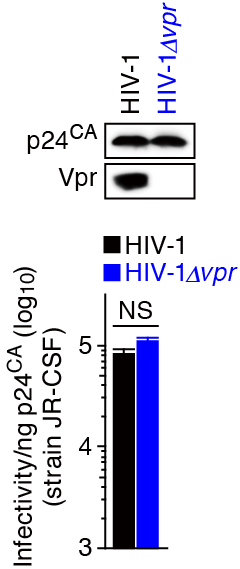

Supplement: Figure S2 — Infectivity of R5 WT and vpr -deficient HIV-1. R5 WT and vpr-deficient HIV-1 (strain JR-CSF) were prepared as described in Materials and Methods. (Top) Western blot analyses of the virions. (Bottom) TZM-bl assay. Prepared virus solutions were inoculated into TZM-bl indicator cells. The infectivities of these viruses were quantified as described in Materials and Methods and were normalized to the amount of p24. The assay was performed in triplicate. NS, no statistical significance. (TIF) [file ppat.1003812.s002.tif]

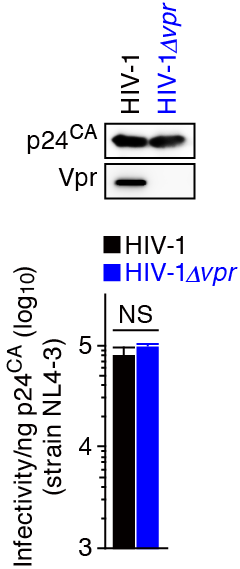

Supplement: Figure S3 — Infectivity of X4 WT and vpr -deficient HIV-1. X4 WT and vpr-deficient HIV-1 (strain NL4-3) were prepared as described in Materials and Methods. (Top) Western blot analyses of the virions. (Bottom) TZM-bl assay. Prepared virus solutions were inoculated into TZM-bl indicator cells. The infectivities of these viruses were quantified as described in Materials and Methods and were normalized to the amount of p24. The assay was performed in triplicate. NS, no statistical significance. (TIF) [file ppat.1003812.s003.tif]

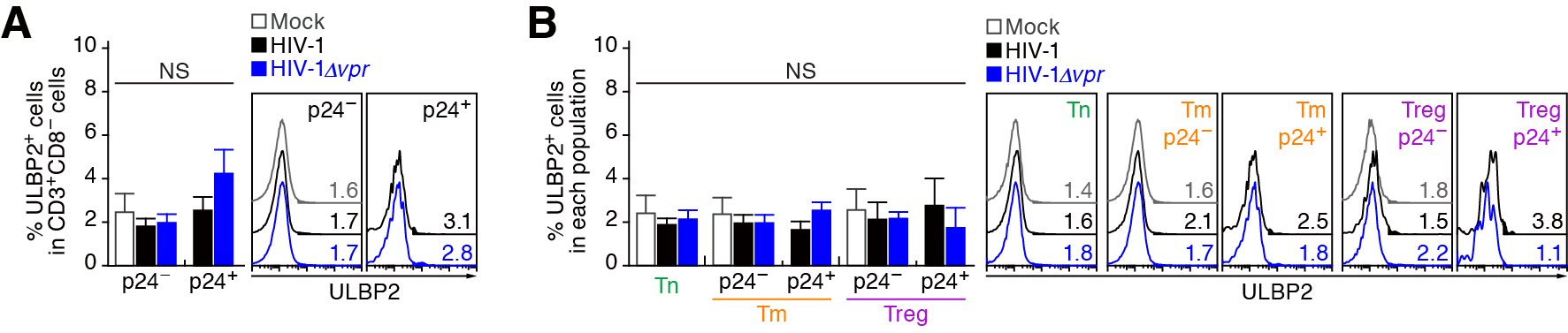

Supplement: Figure S4 — No association of ULBP2 with the Treg depletion observed in WT HIV-1-infected mice. Splenic MNCs of WT HIV-1-infected mice (n = 7), vpr-deficient HIV-1-infected mice (n = 7), and mock-infected mice (n = 7) at 7 dpi were analyzed by flow cytometry using an anti-ULBP2 and an anti-HIV-1 p24 antibodies. The percentages of ULBP2+ cells in CD3+ CD8− cells (A) and in each population (B) are respectively shown. Representative histograms are shown on the right. The numbers in histogram indicate the percentage of active CASP3+ cells in each population. Statistical difference was determined by Welch's t test. NS, no statistical significance. (TIF) [file ppat.1003812.s004.tif]

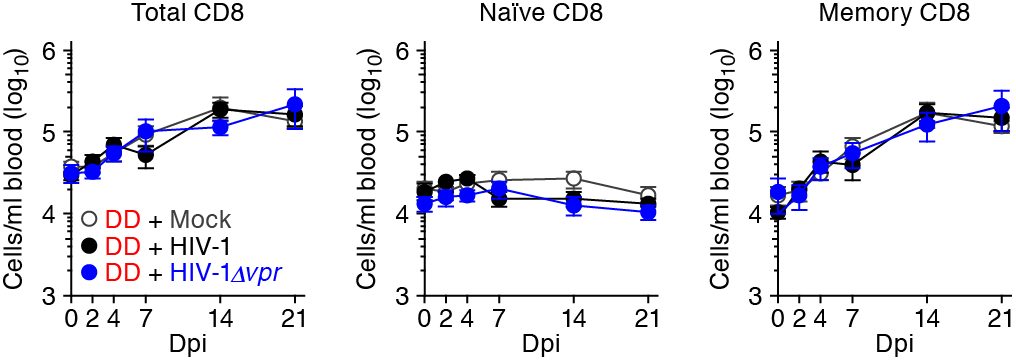

Supplement: Figure S5 — Expansion of memory CD8+ T cells in DD-treated humanized mice. The numbers of total CD8+ T cells (CD45+ CD3+ CD8+ cells), naïve CD8+ T cells (CD45+ CD3+ CD8+ CD45RA+ cells), and memory CD8+ T cells (CD45+ CD3+ CD8+ CD45RA− cells) in the PB of R5 WT HIV-1-infected DD-treated mice (n = 13), R5 vpr-deficient HIV-1-infected DD-treated mice (n = 13), and mock-infected DD-treated mice (n = 8) were routinely quantified by flow cytometry and hematocytometry. (TIF) [file ppat.1003812.s005.tif]
