# Supplementary material for: HIV-1 Vpr Accelerates Viral Replication during Acute Infection by Exploitation of Proliferating CD4+ T Cells In Vivo
Source: PLoS Pathog. 2013 Dec 5;9(12):e1003812. doi: 10.1371/journal.ppat.1003812 (PMC3855622; doi:10.1371/journal.ppat.1003812)
Supplement: Table S1 — Humanized mice used in this study. A full list of the 132 humanized mice used in this study. (PDF) [file ppat.1003812.s006.pdf]

Table S1. Humanized mice used in this study (1 of 4 pages)

Mouse no.	Recipient mouse		Transplanted hHSCs[d]		Inoculated virus[e]	Inoculated age (weeks)	DD treatment
	Lot no.[a]	Sex[b]	Donor lot[c]	No. of cells			
1	66	F	A	200,000	–	14	–
2	66	F	A	200,000	HIV-1	14	–
3	66	M	A	200,000	HIV-1	14	–
4	66	M	A	200,000	–	14	–
5	66	M	A	200,000	HIV-1	14	–
6	66	M	A	200,000	–	14	–
7	66	M	A	200,000	HIV-1	14	–
8	66	M	A	200,000	–	14	–
9	70	F	B	250,000	HIV-1	11	–
10	70	F	B	250,000	HIV-1	11	–
11	70	F	B	250,000	–	11	–
12	70	M	B	250,000	HIV-1	11	–
13	70	M	B	250,000	HIV-1	11	–
14	74	M	A	75,000	–	12	–
15	74	M	A	75,000	–	12	–
16	75	F	A	75,000	–	12	–
17	76	F	A	75,000	–	12	–
18	76	M	A	75,000	–	12	–
19	80	F	C	150,000	–	15	–
20	80	F	C	150,000	HIV-1	15	–
21	80	F	C	150,000	HIV-1*Δvpr*	15	–
22	80	M	C	150,000	HIV-1	15	–
23	80	M	C	150,000	HIV-1	15	–
24	80	M	C	150,000	HIV-1*Δvpr*	15	–
25	80	M	C	150,000	HIV-1*Δvpr*	15	–
26	80	M	C	150,000	HIV-1*Δvpr*	15	–
27	83	F	B	140,000	HIV-1	15	–
28	83	F	B	140,000	HIV-1*Δvpr*	15	–
29	83	F	B	140,000	–	15	–
30	83	F	B	140,000	HIV-1*Δvpr*	15	–
31	84	F	D	140,000	HIV-1	12	–
32	84	F	D	140,000	HIV-1	12	–
33	84	M	D	140,000	HIV-1	12	–
34	84	M	D	140,000	HIV-1*Δvpr*	12	–
35	84	M	D	140,000	HIV-1	12	–
36	84	M	D	140,000	–	12	–
37	84	M	D	140,000	HIV-1	12	–
38	90	F	A	100,000	HIV-1*Δvpr*	12	–
39	90	F	A	100,000	HIV-1	12	–
40	90	F	A	100,000	HIV-1*Δvpr*	12	–
41	90	M	A	100,000	HIV-1	12	–
42	90	M	A	100,000	HIV-1	12	–
43	90	M	A	100,000	HIV-1*Δvpr*	12	–
44	90	M	A	100,000	–	12	–
45	91	F	C	130,000	HIV-1	13	–
46	91	F	C	130,000	HIV-1*Δvpr*	13	–
47	91	F	C	130,000	HIV-1*Δvpr*	16	–
48	91	F	C	130,000	HIV-1	13	–
49	91	F	C	130,000	HIV-1*Δvpr*	13	–
50	91	F	C	130,000	HIV-1*Δvpr*	16	–

(Continued on following page)

Table S1. Humanized mice used in this study (2 of 4 pages)

Mouse no.	Recipient mouse Lot no.[a]	Recipient mouse Sex[b]	Transplanted hHSCs[d] Donor lot[c]	Transplanted hHSCs[d] No. of cells	Inoculated virus[e]	Inoculated age (weeks)	DD treatment
51	91	F	C	130,000	HIV-1	13	–
52	91	F	C	130,000	HIV-1 *Δvpr*	13	–
53	91	M	C	130,000	HIV-1 *Δvpr*	16	–
54	91	M	C	130,000	HIV-1 *Δvpr*	13	–
55	92	F	B	180,000	HIV-1	16	–
56	92	F	B	180,000	HIV-1	13	–
57	92	F	B	180,000	HIV-1 *Δvpr*	13	–
58	92	F	B	180,000	HIV-1	16	–
59	92	M	B	180,000	HIV-1 *Δvpr*	16	–
60	92	M	B	180,000	HIV-1 *Δvpr*	16	–
61	94	F	E	180,000	HIV-1	13	–
62	94	F	E	180,000	HIV-1 *Δvpr*	13	–
63	94	F	E	180,000	HIV-1	13	–
64	94	F	E	180,000	HIV-1 *Δvpr*	13	–
65	94	M	E	180,000	HIV-1	13	–
66	94	M	E	180,000	HIV-1 *Δvpr*	13	–
67	94	M	E	180,000	HIV-1	13	–
68	94	M	E	180,000	HIV-1 *Δvpr*	13	–
69	97	M	B	160,000	–	12	–
70	98	F	B	140,000	–	12	–
71	98	F	B	140,000	–	12	–
72	99	F	A	84,000	HIV-1	20	–
73	99	F	A	84,000	HIV-1 *Δvpr*	20	–
74	99	F	A	84,000	HIV-1	20	–
75	100	F	A	130,000	HIV-1 *Δvpr*	18	–
76	101	F	B	160,000	HIV-1	14	–
77	101	M	B	160,000	HIV-1 *Δvpr*	14	–
78	102	F	E	180,000	HIV-1	14	–
79	102	M	E	180,000	HIV-1 *Δvpr*	14	–
80	102	M	E	180,000	HIV-1	14	–
81	105	F	F	140,000	–	14	–
82	106	F	G	120,000	–	14	–
83	106	M	G	120,000	–	14	–
84	108	M	H	240,000	–	14	–
85	133	F	I	200,000	HIV-1	17	–
86	133	F	I	200,000	HIV-1	17	–
87	133	F	I	200,000	HIV-1	17	–
88	133	M	I	200,000	HIV-1	17	–
89	134	F	I	150,000	HIV-1 *Δvpr*	16	–
90	134	F	I	150,000	HIV-1 *Δvpr*	16	–
91	134	F	I	150,000	HIV-1 *Δvpr*	16	–
92	134	M	I	150,000	HIV-1 *Δvpr*	16	–
93	134	M	I	150,000	HIV-1	16	–

(Continued on following page)

Table S1. Humanized mice used in this study (3 of 4 pages)

Mouse no.	Recipient mouse Lot no.[a]	Recipient mouse Sex[b]	Transplanted hHSCs[d] Donor lot[c]	Transplanted hHSCs[d] No. of cells	Inoculated virus[e]	Inoculated age (weeks)	DD treatment
94	114	F	H	180,000	HIV-1	15	+
95	114	F	H	180,000	HIV-1 *Δvpr*	15	+
96	114	F	H	180,000	HIV-1 *Δvpr*	15	+
97	114	M	H	180,000	HIV-1	15	+
98	114	M	H	180,000	HIV-1 *Δvpr*	15	+
99	114	M	H	180,000	HIV-1	15	+
100	115	F	H	190,000	HIV-1 *Δvpr*	15	+
101	115	F	H	190,000	HIV-1 *Δvpr*	15	+
102	115	F	H	190,000	HIV-1	15	+
103	115	F	H	190,000	HIV-1 *Δvpr*	15	+
104	115	F	H	190,000	HIV-1	15	+
105	115	F	H	190,000	HIV-1 *Δvpr*	15	+
106	115	F	H	190,000	HIV-1	15	+
107	115	M	H	190,000	HIV-1	15	+
108	122	F	G	110,000	–	17	+
109	122	F	G	110,000	–	17	+
110	122	M	G	110,000	–	17	+
111	122	M	G	110,000	–	17	+
112	123	F	G	110,000	–	17	+
113	123	F	G	110,000	–	17	+
114	123	M	G	110,000	HIV-1 *Δvpr*	17	+
115	123	M	G	110,000	HIV-1	17	+
116	130	F	J	180,000	–	14	+
117	130	F	J	180,000	–	14	+
118	130	F	J	180,000	HIV-1	14	+
119	130	F	J	180,000	HIV-1	14	+
120	130	M	J	180,000	HIV-1 *Δvpr*	14	+
121	130	M	J	180,000	HIV-1 *Δvpr*	14	+
122	131	F	I	120,000	–	13	+
123	131	F	I	120,000	–	13	+
124	137	F	K	110,000	HIV-1 *Δvpr*	16	+
125	137	F	K	110,000	–	16	+
126	137	F	K	110,000	–	16	+
127	137	F	K	110,000	–	16	+
128	137	F	K	110,000	HIV-1	16	+
129	137	M	K	110,000	HIV-1 *Δvpr*	16	+
130	137	M	K	110,000	HIV-1 *Δvpr*	16	+
131	137	M	K	110,000	HIV-1	16	+
132	137	M	K	110,000	HIV-1	16	+

(Continued on following page)

Table S1. Humanized mice used in this study (4 of 4 pages)

Mouse no.	Recipient mouse		Transplanted hHSCs[d]		Inoculated virus[f]	Inoculated age (weeks)	DD treatment
	Lot no.[a]	Sex[b]	Donor lot[c]	No. of cells			
133	159	F	L	130,000	HIV-1	16	–
134	159	F	L	130,000	HIV-1	16	–
135	159	F	L	130,000	–	16	–
136	159	F	L	130,000	–	16	–
137	159	M	L	130,000	HIV-1	16	–
138	159	M	L	130,000	HIV-1 *Δvpr*	16	–
139	160	F	M	120,000	–	14	–
140	160	F	M	120,000	HIV-1 *Δvpr*	14	–
141	160	F	M	120,000	HIV-1	14	–
142	160	F	M	120,000	–	14	–
143	160	F	M	120,000	–	14	–
144	160	M	M	120,000	HIV-1 *Δvpr*	14	–
145	160	M	M	120,000	HIV-1	14	–
146	161	F	N	100,000	HIV-1 *Δvpr*	18	–
147	162	F	N	140,000	HIV-1	17	–
148	162	M	N	140,000	–	17	–
149	162	M	N	140,000	HIV-1	17	–
150	162	M	N	140,000	HIV-1 *Δvpr*	17	–
151	163	F	N	100,000	–	17	–
152	163	F	N	100,000	HIV-1 *Δvpr*	17	–
153	163	F	N	100,000	HIV-1	17	–
154	163	F	N	100,000	HIV-1	17	–
155	163	F	N	100,000	HIV-1 *Δvpr*	17	–
156	163	M	N	100,000	–	17	–
157	164	F	N	90,000	HIV-1	15	–
158	164	F	N	90,000	HIV-1 *Δvpr*	15	–
159	164	M	N	90,000	HIV-1	15	–
160	165	M	L	170,000	HIV-1	14	–
161	166	F	N	120,000	HIV-1	12	–
162	166	M	N	120,000	HIV-1 *Δvpr*	12	–
163	166	M	N	120,000	HIV-1 *Δvpr*	12	–
164	166	M	N	120,000	HIV-1 *Δvpr*	12	–

[a] Thirty eight lots of newborn NOG mice were used for the recipient.
[b] M, male; F, female.
[c] NOG-hCD34 mice were reconstituted with one of 14 donors.
[d] hHSCs, human $CD34^+$ hematopoietic stem cells.
[e] Strain JR-CSF (CCR5-tropic).
[f] Strain NL4-3 (CXCR4-tropic).
