# Supplementary material for: HIV-1 Vpr Accelerates Viral Replication during Acute Infection by Exploitation of Proliferating CD4+ T Cells In Vivo
Source: PLoS Pathog. 2013 Dec 5;9(12):e1003812. doi: 10.1371/journal.ppat.1003812 (PMC3855622; doi:10.1371/journal.ppat.1003812)
Supplement: Table S2 — Antibodies used in flow cytometry analyses. A full list of antibodies used in this study. (PDF) [file ppat.1003812.s007.pdf]

Table S2. Antibodies used in flow cytometry analyses.

Antigen	Clone	Fluorophore	Source
CD3	HIT3a	APC	Biolegend
CD3	HIT3a	APC-Cy7	Biolegend
CD4	RPA-T4	APC	Biolegend
CD4	RPA-T4	PE-Cy7	Biolegend
CD8	DK25	FITC	Dako
CD8	DK25	APC	Dako
CD8	HIT8α	PE-Cy7	Biolegend
CD25	BC96	APC	eBioscience
CD38	HIT2	APC	Biolegend
CD45	HI30	PE	Biolegend
CD45RA	HI100	FITC	Biolegend
CD45RA	HI100	PerCP-Cy5.5	Biolegend
CD127	A019D5	APC	Biolegend
CASP3	C92-605	PE	BD Biosciences
CCR5	2D7	FITC	BD Biosciences
CTLA4	BNI3	APC	BD Biosciences
CXCR4	12G5	PE	BD Biosciences
FOXP3	236A/E7	APC	Biolegend
FOXP3	236A/E7	Pacific blue	Biolegend
FOXP3	236A/E7	PE	Biolegend
MKI67	B56	PE	BD Biosciences
ULBP2	–	PE	R&D systems
HIV-1 p24	2C2	FITC	Provided by Yuetsu Tanaka.*

* Okuma K, Tanaka R, Ogura T, et al. Interleukin-4-transgenic hu-PBL-SCID mice: a model for the screening of antiviral drugs and immunotherapeutic agents against X4 HIV-1 viruses. J Infect Dis. 2008;197:134-141.
